# Matrix Metalloproteinase-10 in Kidney Injury Repair and Disease

**DOI:** 10.3390/ijms23042131

**Published:** 2022-02-15

**Authors:** Xiaoli Sun, Youhua Liu

**Affiliations:** 1 State Key Laboratory of Organ Failure Research, National Clinical Research Center of Kidney Disease, Division of Nephrology, Nanfang Hospital, Southern Medical University, Guangzhou 510515, China; sunxiaoli233@163.com; 2 Department of Pathology, School of Medicine, University of Pittsburgh, S405 Biomedical Science Tower, 200 Lothrop Street, Pittsburgh, PA 15261, USA

**Keywords:** MMP-10, acute kidney injury, chronic kidney disease, renal fibrosis, proteinuria, HB-EGF, ZO-1

## Abstract

Matrix metalloproteinase-10 (MMP-10) is a zinc-dependent endopeptidase with the ability to degrade a broad spectrum of extracellular matrices and other protein substrates. The expression of MMP-10 is induced in acute kidney injury (AKI) and chronic kidney disease (CKD), as well as in renal cell carcinoma (RCC). During the different stages of kidney injury, MMP-10 may exert distinct functions by cleaving various bioactive substrates including heparin-binding epidermal growth factor (HB-EGF), zonula occludens-1 (ZO-1), and pro-MMP-1, -7, -8, -9, -10, -13. Functionally, MMP-10 is reno-protective in AKI by promoting HB-EGF-mediated tubular repair and regeneration, whereas it aggravates podocyte dysfunction and proteinuria by disrupting glomerular filtration integrity via degrading ZO-1. MMP-10 is also involved in cancerous invasion and emerges as a promising therapeutic target in patients with RCC. As a secreted protein, MMP-10 could be detected in the circulation and presents an inverse correlation with renal function. Due to the structural similarities between MMP-10 and the other MMPs, development of specific inhibitors targeting MMP-10 is challenging. In this review, we summarize our current understanding of the role of MMP-10 in kidney diseases and discuss the potential mechanisms of its actions.

## 1. Introduction

Matrix metalloproteinase-10 (MMP-10), also known as stromelysin-2 or tansin-2, is a zinc-dependent endopeptidase. It belongs to the stromelysin subfamily of MMPs, along with MMP-3 and MMP-11 [1,2]. As a proteolytic enzyme, MMP-10 is secreted outside of the cell as a zymogen and subsequently activated by diverse proteinases [3]. Intriguingly, activated MMP-10 can also be detected in the nuclei and cytosol, suggesting the possibility of additional biological functions of this proteinase [4,5,6]. The expression of MMP-10 is hardly detectable in developing or normal adult tissues including kidney, but it is markedly increased after tissue injury [7,8,9,10]. Such an induction of MMP-10 under pathological conditions indicates that this proteinase may play a critical role in the host response to environmental insults. Dysregulation of MMP-10 is implicated in the pathogenesis of various disorders including kidney diseases, tumorigenesis, atherosclerosis, and cardiovascular diseases.

As a matrix-degrading enzyme, MMP-10 is primarily involved in tissue remodeling by digesting various extracellular matrix (ECM) components such as types III, IV and V collagens, elastin, gelatin, proteoglycans, and fibronectin [11]. The inability to digest type I collagen is the main feature that distinguishes stromelysins from collagenases. Recent studies, however, have uncovered that MMP-10 also participates in regulating a variety of biological processes by cleaving numerous bioactive molecules on the cell surface in the kidney [7,12]. New substrates of MMP-10 have increasingly been identified, such as heparin binding-epidermal growth factor (HB-EGF) and zonula occludens-1 (ZO-1), among others [7,12,13,14]. Functionally, MMP-10 can be viewed as a double-edged sword in renal pathophysiology under different circumstances. While MMP-10 is reno-protective in acute kidney injury (AKI) by promoting HG-EGF-mediated injury repair and regeneration, it also contributes to the pathogenesis of chronic kidney diseases (CKD). MMP-10 as a secreted protein can be found in the blood circulation and its levels inversely correlate with renal function in CKD patients.

In this article, we highlight recent advances in our understanding of the biology of MMP-10 and its regulation in diseased kidneys. We also review the role of MMP-10 in the evolution of AKI and CKD and discuss the potential mechanisms of its action.

## 2. Biology of MMP-10

MMP-10 belongs to a large family of MMPs consisting of 25 known members [15]. MMP-10 shares common structural motifs with other MMPs. MMP-10 activities are modulated on several levels including transcription, pro-enzyme activation, or inhibition by its inhibitors.

### 2.1. Structure of MMP-10

**Figure 1:**
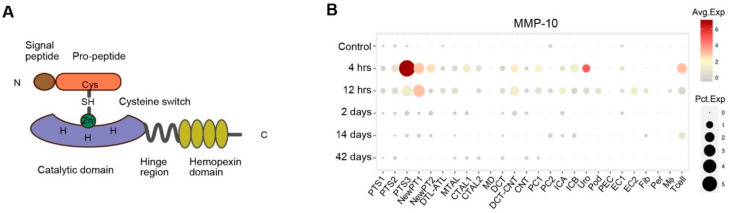
Domain structure of MMP-10 and its expression pattern after acute kidney injury. (**A**) The pre-pro-MMP10 is principally composed of four domains: N-terminal signal peptide, pro-peptide domain, catalytic domain, as well as hemopexin domain. The interaction between hydrosulphonyl (SH) of the cysteine residue (Cys) located in the pro-peptide, and the zinc ion (Zn) situated in the catalytic domain constitutes the “cysteine switch”, which controls the enzyme activity. (**B**) The dynamics and expression pattern of MMP-10 after acute kidney injury (AKI). Bubble diagram displays MMP-10 expression in different types of kidney cells at various time points following ischemia-reperfusion injury (IRI) based on single cell RNA sequencing results in the Kidney Interactive Transcriptomics database (http://humphreyslab.com/SingleCell, accessed on 20 December 2021) [20]. PT-S1, S1 segment of proximal tubule; PT-S2, S2 segment of proximal tubule; PT-S3, S3 segment of proximal tubule; DTL, descending limb of loop of Henle; MTAL, thick ascending limb of loop of Henle in medulla; CTAL, thick ascending limb of loop of Henle in cortex; MD, macula densa; DCT, distal convoluted tubule; CNT, connecting tubule; PC, principle cells; ICA, type A intercalated cells of collecting duct; ICB, type B intercalated cells of collecting duct; Uro, urothelium; Pod, podocytes; PEC, parietal epithelial cells; EC, endothelial cells; Fib, fibroblasts; Per, pericytes; Mø, macrophages; Avg Exp, average expression; Pct Exp, percent expressed.

As a secreted protein, MMP-10 is primarily synthesized as the 476 amino acid zymogen comprised of four domains: 17 amino acid N-terminal signal peptide, 81 amino acid pro-peptide domain, 165 amino acid catalytic domain, and 187 amino acid hemopexin domain [16,17,18] (Figure 1A). Interestingly, recent studies reveal that a nuclear localization sequence (NLS) might exist in the C-terminal, which contributes MMP-10 to be localized and targeted to the nucleus [5,6,19].

The pro-peptide contains a cysteine switch motif [21], an intramolecular complex formed between the sulfhydryl group of the cysteine residue in the pro-peptide domain and the essential zinc ion located in the catalytic domain. The cysteine switch motif blocks the active site and maintains the precursor structure [16]. Consequently, disruption of this motif can release active enzyme [22].

Two zinc ions (one catalytic and one structural) and three calcium ions are indispensable for the catalytic site. Three histidines from the zinc binding motif in the catalytic domain coordinate with catalytic zinc and constitute the catalytic site [23]. Importantly, the substrate specificity is predominantly determined by the S1′ hydrophobic pocket which is adjacent to the catalytic site [24].

The hemopexin domain with four hemopexin-like repeats presents four-bladed propeller folds which links with the catalytic domain by a flexible hinge region. In addition to regulating protein–protein interaction, the hemopexin domain could play an auxiliary role in helping substrate binding at catalytic sites [25].

### 2.2. Transcriptional Regulation of MMP-10

In humans, the *MMP-10* gene is mapped to chromosomes at 11q22.2. A set of various *trans*-activators can modulate the expression of *MMP-10* by regulating certain *cis*-elements, including TATA boxes, activated protein-1 (AP-1) binding site, polyoma enhancer activator 3 (PEA3)-binding site, β-catenin/T-cell factor 4 (TCF4), and nuclear factor-ĸB (NF-ĸB) [26,27,28]. The interplay of a variety of *cis*-acting elements with cognate transcription factors confers the response to specific cues in various tissues and cell types. Moreover, DNA hypomethylation can also modulate transcriptional activity on the *MMP-10* promoter in a cell-specific manner [29].

NF-κB and AP-1 are widely activated in all kinds of kidney disorders and participate in the progression of nephropathies [30]. Transforming growth factor-β (TGF-β), a powerful profibrotic cytokine, may transactivate *MMP-10* by a specific interaction between the Smad proteins and AP-1 [31]. Furthermore, another study shows that *MMP-10* expression is regulated by TGF-β through mediating activation of myocyte enhancer factor 2A (MEF2A) and downregulating class IIα histone deacetylases (HDACs) [32]. In addition, biomechanical stretching also induces *MMP-10* expression by mediating NF-ĸB activation in podocytes in vitro [33].

Bioinformatics analysis shows that there are two putative TCF/LEF-binding sites (TBS) in the promoter region of *MMP-10* [34]. In various kinds of nephropathies, canonical Wnt/β-catenin signaling is activated and plays a pivotal role in mediating kidney injury, repair and pathogenesis [35,36,37]. Recent studies indicate that Wnt7a induces MMP-10 expression through the canonical β-catenin pathway [34]. Furthermore, overexpression of Wnt5b in vitro upregulates MMP-10 expression as well [38]. As Wnt/β-catenin is activated in all kinds of AKI and CKD examined, these findings suggest a widespread induction of MMP-10 in various kidney disorders.

### 2.3. Posttranscriptional Activation of MMP-10

MMP-10 is commonly regarded as an extracellular proteinase. However, recent finding shows that MMP-10 is also activated inside the cell and involved in the pathogenesis of diseases [6]. The mechanism by which MMP-10 enters cells has not been fully elucidated. During the activation, pro-MMP-10 is enzymatically cleaved, and its conformation is modified. Pro-MMP-10 is secreted into the extracellular space after the N-terminal signal peptide is removed and can be subsequently activated by various factors [16]. In extracellular milieu, the His81-Phe82 bond in the pro-peptide is often cleaved by other proteolytic enzymes, such as plasma kallikrein, trypsin, neutrophil elastase, or cathepsin G [3,17]. As such, this procedure contributes to the conversion of 56 kDa latent MMP-10 to mature active forms, which are 47 kDa and 24 kDa, respectively [21]. Along with this, the cysteine residue is dissociated from catalytic zinc that leads to cysteine-switch activation, and the catalytic site is exposed [22].

It has been shown that pro-MMP-10 is labile and can undergo autolysis, leading to the production of mature MMP-10 [21]. Under nondenaturing conditions, MMP-10 can also be activated through conformational change without cleavage of the pro-peptide after exposure to SDS [16]. Furthermore, organomercurials may interact with the unpaired thiol group in the pro-peptide, resulting in autolytic cleavages and the opening of the cysteine switch [16].

### 2.4. Inhibitors of MMP-10

Inhibitors of MMP-10 are composed of two classes: endogenous and synthetic. Tissue inhibitors of metalloproteinases (TIMPs), consisting of TIMP1~4, are secreted proteins and function against multiple secreted or membrane-anchored metalloproteinases [39]. In addition, TIMPs play a pivotal role in modulating the influence of the extracellular environment on cell phenotype [40]. It has been demonstrated that each of the TIMPs can inhibit MMP-10 activity by forming a complex in a 1:1 stoichiometry [41,42,43]. In spite of substantial structural differences between the TIMPs, most of the flexible loops of the MMP-10 catalytic domain are locked into nearly indistinguishable conformations upon TIMP binding [42]. Disrupting the balance between MMP-10 and TIMPs will contribute to a broad spectrum of devastating diseases.

Although TIMPs can be considered as MMP-10 endogenous inhibitors, their actions are not merely on MMP-10 [39]. The development of a specific MMP-10 inhibitor with a pure inhibitory spectrum is urgent. Structurally, MMP-10 clearly resembles other MMPs, especially MMP-3, as they share 78% overall amino acid sequence identity and 86% sequence identity in their catalytic domain in humans. This makes it difficult to develop a selective and potent MMP-10 inhibitor [17,23]. Nevertheless, there are several studies reporting that selective dual MMP-10/-13 inhibitors possess a clean inhibition profile and outstanding affinity for MMP-10 [44,45]. Furthermore, MMP-10 activity can be regulated by increased or diminished expression at the transcriptional or translational levels. Synthetic inhibitors generally contain a chelating group that binds the catalytic zinc atom at the MMP active site. Other inhibitors interact with various binding pockets on the MMP of interest resulting in specific inhibitory potentials for the given MMP. In addition, single-domain antibodies devoid of a light chain and CH1 region emerge as attractive inhibitors [46]. A recent study reported a single-domain antibody, H3, which can selectively inhibit MMP-10 activity by binding to its active site [47]. Further studies on developing specific inhibitors of MMP-10 are needed to provide a novel therapeutic strategy for various diseases with hyperactive MMP-10.

## 3. MMP-10 Expression in the Kidney

Contrary to its weak staining in normal human kidney tubular cells [48], MMP-10 is scarcely expressed in normal murine kidneys. It is substantially induced under pathological conditions [7,12]. Consistently, MMP-10-/- mice and transgenic mice with podocyte-specific expression of MMP-10 do not display overt renal abnormality [7,49]. These findings indicate that MMP-10 is not required for murine kidney structure and function under normal physiological conditions. However, overexpression of MMP-10 renders transgenic mice susceptible to developing more severe podocytopathy after injury [7].

In response to diverse insults, MMP-10 can be induced in various cells, including the renal tubular epithelium, glomerular podocytes, juxtaglomerular apparatus, as well as renal cell carcinoma [7,12,48,50]. Table 1 shows the expression and localization of MMP-10 in various kidney disorders.

### 3.1. AKI

Induction of MMP-10 is a common pathological finding in various models of AKI. Studies show that MMP-10 protein is substantially upregulated in various animal models of AKI, including ischemia-reperfusion injury (IRI), cisplatin-induced nephrotoxic AKI and glycerol-induced rhabdomyolysis-associated AKI [12,51]. Notably, the mRNA level of *MMP-10* is induced as early as 4 h and sustained at least to 48 h after IRI [12]. Data mining from the Kidney Interactive Transcriptomics database created by single-nuclei RNA-sequencing reveals the dynamics and landscape of *MMP-10* expression in mouse AKI induced by IRI (Figure 1B) (http://humphreyslab.com/SingleCell, accessed on 20 December 2021). Furthermore, proximal tubules, especially the S3 segment, are the main cell population that express *MMP-10* following IRI (Figure 1B) [20]. Immunohistochemical staining reveals that MMP-10 is strongly stained in the injured area of the kidney and predominantly localizes in the renal tubular epithelium [12,51]. The mechanism underlying MMP-10 induction in AKI, as well as the triggers for induction, remains unclear. Notably, the canonical Wnt/β-catenin signaling, which protects the kidney from AKI [56], is activated in this setting. This may account for the regulatory mechanism of MMP-10 expression in response to AKI.

### 3.2. CKD

MMP-10 can also be detected in the glomerular podocytes of CKD patients with diabetic kidney disease (DKD), IgA nephropathy (IgAN), and focal segmental glomerulosclerosis (FSGS) [7]. Consistent with this finding, MMP-10 protein is not only induced significantly in adriamycin (ADR) mice in a time-dependent fashion, but is also upregulated in other proteinuric CKD models, such as diabetic *db/db* mice, remnant kidney model after 5/6 nephrectomy (5/6NX), Alport mice, CD151 null mice and α-actinin-4 knockout mice, which are a Alport syndrome model and a model of FSGS, respectively [7,33,50,55]. Furthermore, compared with the controls, zymographic analysis also reveals an increase in MMP-10 proteolytic activity in the kidney after ADR [7]. In experimental models, MMP-10 is not only localized in glomerular podocytes but also in the juxtaglomerular apparatus [7,50,53].

### 3.3. RCC

Upregulation of *MMP-10* is also common in numerous cancers [57]. In patients with renal cell carcinoma (RCC), immunohistochemical staining reveals that induction of MMP-10 is primarily detected in tubular cancer cell cytoplasm [48]. Meanwhile, MMP-10 expression exhibits a tight correlation with the grade and pT stage. Cancer cells of the invasive front strongly express MMP-10, and the majority of sarcomatous cancer cells shows moderate or strong intensity [48]. As such, sarcomatous change represents a transformation to a higher-grade malignancy and portends a worse prognosis in renal cell carcinoma [58]. Consequently, these findings suggest that MMP-10 is relevant to RCC invasion. 

MMP-10 appears to be the target of diverse factors involved in RCC invasion. In favor of this notion, ghrelin, a peptide hormone, binds to the ghrelin receptor and results in MMP-10 expression by upregulating Aurora A, which results in RCC invasion [59]. In vitro, MMP-10 is upregulated by IL-1β, mechanistically due to activation of transcription factor CCAAT enhancer binding protein β (CEBPβ) [60]. In addition, there is a study reporting that activation of *MMP-10* might be involved in the secreted frizzled-related protein 1 (SFRP1)-caused cell invasion in metastatic RCC [61].

## 4. Role of MMP-10 in Kidney Disease 

A plethora of evidence suggests that MMP-10 is a mixed blessing, which plays a crucial role in kidney repair/regeneration and the pathogenesis of CKD. The actions of MMP-10 result in either beneficial or detrimental consequences, depending on the specific setting. In response to AKI, MMP-10 acts as a renal-protective factor [12]. However, sustained activation of MMP-10 after severe or repeated injury emerges as a stimulant in the progression of CKD [7]. Moreover, the expression of MMP-10 in RCC can be harnessed as a potential therapeutic target to inhibit RCC tumor progression [48]. Table 2 summarizes the distinct role of MMP-10 in different kidney disorders.

### 4.1. Acute Kidney Injury

AKI, characterized by an abrupt loss of kidney function, is highly prevalent and related to high morbidity and mortality [64,65]. In aging kidney, the structural and functional changes increase the risk for AKI [66,67]. Persistent geriatric AKI is independently associated with a higher risk of 90-day mortality [68]. As a rule, in the case where conservative interventions are not effective, renal replacement therapy (RRT) is advisable [69]. However, suitable time of initiating RRT remains controversial. There is a study showing that serum MMP-10 levels serve as an important predictor of the necessity of emergency RRT in non-diabetic geriatric patients with AKI [62]. Nevertheless, because of the limited number of patients in this study, careful follow-up evaluation with a larger sample size is needed to confirm this finding.

In experimental animal models, expression of exogenous MMP-10 protects renal tubular epithelial cells from AKI, characterized by inhibiting tubular cells apoptosis, promoting their proliferation and regeneration [12]. These findings appear to be compatible with the notion that MMP-10 promotes tissue repair and regeneration in other organs such as liver, bone, and colon [4,9,70,71]. Consistent with that, ectopic expression of MMP-10 preserves renal function, and knockdown of endogenous MMP-10 deteriorates AKI after IRI [12]. Taken together, these findings support a protective role of MMP-10 in AKI. 

### 4.2. Diabetic Kidney Disease

DKD is a major complication of diabetes mellitus (DM) and the most common cause of end stage renal disease (ESRD) [72]. It has been shown that increased incidence of diabetic nephropathy leads to an elevation of diabetic vascular complications mortality [73]. As a secretory protein, MMP-10 can be detected in human serum. Numerous studies show that serum MMP-10 is significantly elevated in type 1 and type 2 diabetic patients [49,50,74]. Among type 1 diabetic patients, those with high serum MMP-10 levels exhibit a higher risk of developing diabetic nephropathy [49]. Similarly, there is a tight association between the degree of albuminuria and plasma levels of MMP-10 [74], indicating a detrimental role of MMP-10. Consistent with this view, in a streptozotocin (STZ)-induced murine diabetes model, absence of MMP-10 partially protects kidney from diabetes-induced renal injury, manifested by improved renal function, less expansion of the mesangial matrix, as well as reduced macrophage influx [49].

The elevated circulating levels of MMP-10 present an inverse correlation with estimated glomerular filtration rate (eGFR), which is observed even at early stages of type 2 diabetes [50]. This is in harmony with animal experiments that *MMP-10* expression is upregulated in mice from the earliest stages of DKD [50]. These studies suggest that MMP-10 might be involved in the onset of diabetic nephropathy, although the underlying mechanism remains to be elucidated.

### 4.3. Nondiabetic Glomerular Disease

MMP-10 is involved in the development and progression of nondiabetic glomerular diseases. In mouse ADR models, glomerular injury and kidney fibrosis are exacerbated by ectopic expression of exogenous MMP-10 [7]. These effects can also be observed after ADR injection in transgenic mice with podocyte-specific expression of MMP-10 [7]. Therefore, MMP-10 serves as a detrimental regulator of podocyte injury and proteinuria in experimental models. As anticipated, knockdown of endogenous MMP-10 following ADR protects the kidney against proteinuria and glomerular sclerosis [7].

Mounting evidence shows that levels of serum pro-MMP-10 are not only elevated in patients with CKD, but also correlate with the severity of kidney dysfunction, manifested by positive association with serum creatinine, cystatin C, and proteinuria [7,50,75]. In addition, serum MMP-10 levels exhibit the capacity to distinguish systemic lupus erythematosus (SLE) from healthy controls, which predicts organ damage and lupus nephritis in SLE patients [76,77]. However, more large-scale studies are needed to confirm whether MMP-10 can be a biomarker for predicting and monitoring kidney function.

The kidney is the most important target organ affected by sustained hypertension. There is a study suggesting that MMP-10 might be involved in the development of hypertensive nephropathy once essential hypertension has been established, since circulating MMP-10 is elevated in ESRD patients, when compared with normotensive and hypertensive groups [63]. Additionally, plentiful studies suggest that MMP-10 has the potential to drive the progression of atherosclerosis [78,79] and plays a role in vascular remodeling [3,80,81]. Indeed, the serum MMP-10 level is independently associated with carotid atherosclerosis [82], consistent with the notion that circulating MMP-10 level is an independent risk factor for atherosclerosis in CKD patients [75].

### 4.4. Alport Glomerular Disease

Alport syndrome affects glomerular, cochlear, and ocular basement membrane and is a genetically and phenotypically heterogeneous disorder, attributing to variants in the collagen IV genes *COL4A3*, *COL4A4*, and *COL4A5* [83]. Mutations of collagen IV, a major component of the glomerular basement membrane (GBM), lead to progressive impairment of GBM, giving rise to characteristic basket-weave appearance, accompanied by accelerated podocyte detachment from the GBM [84,85]. As the disease progresses, defective GBM and loss of podocytes eventually lead to glomerular sclerosis and obsolescence [86,87]. It has been shown that MMP-10 is upregulated as early as 3-weeks in FVB/N CD151-/- mice [53], suggesting an underlying function of MMP-10 in the early stage of Alport syndrome. 

In vivo, treatment of Alport mice with either TEA226, a small molecule inhibitor of focal adhesion kinase (FAK), or PAT-1251, a small molecule inhibitor for lysyl oxidase-like 2 (LOXL2), can abolish MMP-10 expression, resulting in amelioration of mesangial expansion, improvement of GBM architecture, alleviation of kidney fibrosis and monocytic infiltration, as well as restoration of renal function by reducing blood urea nitrogen (BUN) and albuminuria [33,52]. These results establish a link between MMP-10 and Alport glomerular disease, although direct causative proof of MMP-10 is still absent. 

In the light of recent studies, it is conceivable that FAK is a potent regulator for MMP-10 expression, at least in Alport syndrome. In fact, accumulation of laminin α2 in the GBM is a common feature of Alport mice [33]. It has been shown that laminin α2 is able to induce *MMP-10* mRNA expression by mediating FAK activation in podocytes both in vivo and in vitro [33]. Of note, biomechanical stress acts as a fundamental driver of glomerular pathogenesis in Alport syndrome [87]. Studies have shown that biomechanical stretching results in the induction of *MMP-10* mRNA by FAK activation in podocytes [33]. Furthermore, in Alport mice, inhibition of LOXL2 significantly prevents accumulation of laminin α2 and reduces *MMP-10* mRNA in glomeruli [52]. Accordingly, it is tempting to speculate that LOXL2 upregulates MMP-10 expression in Alport mice possibly through promoting laminin α2 accumulation in GBM, which results in FAK activation.

### 4.5. Kidney Cancer

RCC, the most common malignant tumor of the urinary system, is among the 10 most common cancers in the United States [88,89]. Numerous studies show that knockdown of MMP-10 expression or blocking MMP-10 activity significantly reduces tumor cell invasion in vitro [38,90]. Consistently, the connection between MMP-10 and RCC invasion has been explored as well. Multivariate analysis model reveals that MMP-10 expression is an independent factor of tumor invasion in RCC patients [48]. In human 786-0 VHL null RCC cell lines, IL-1β-induced RCC tumor cell invasion is, at least partially, dependent on MMP-10 [60]. Furthermore, the silence of MMP-10 decreases ghrelin-induced metastatic renal adenocarcinoma cell invasion [59].

MMP-10 is upregulated in various cancers and is involved in tumor metastasis [91,92]. However, contrary to these findings, some studies show that MMP-10 expression is not associated with metastasis, tumor growth, or cancer cell proliferation in patients with RCC [48]. Despite that, MMP-10 expression is not an independent factor by multivariate analysis, it is a predictor of poor survival by univariate analysis [48]. A more recent study confirms that a high level of MMP-10 in renal cancer patients is associated with poor survival [59].

## 5. Mechanism of MMP-10 Action in Kidney Disease

**Figure 2:**
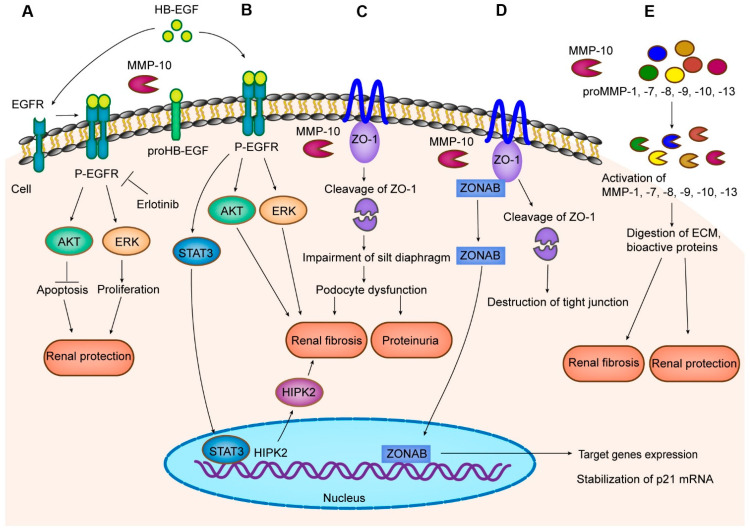
Biological roles of MMP-10 and the mechanisms of its action in kidney diseases. (**A**) MMP-10 secreted by tubular cells elicits reno-protective activities in response to AKI by cleaving HB-EGF and activating EGFR signaling. (**B**) Sustained activation of EGFR due to the release of active HB-EGF by MMP-10, however, leads to tubulointerstitial fibrosis. (**C**) MMP-10 proteolytically degrades ZO-1, a key component of silt diaphragm, thereby impairing podocyte integrity and promoting proteinuria and glomerulosclerosis in CKD. (**D**) Furthermore, MMP-10-mediated loss of ZO-1 results in nuclear translocation of ZONAB, which represses differentiation of proximal tubular epithelia cells and promotes cellular senescence. (**E**) MMP-10 may promote renal repair and disease through activating pro-MMP-1, -7, -8, -9, -10, -13. Different colors of the circles represent different pro-MMPs, respectively. EGFR, epidermal growth factor receptor; ERK, extracellular signal-regulated kinase; AKT, Akt kinase, also known as protein kinase B; STAT3, signal transducer and activator of transcription 3; ZO-1, zonula occludens-1; ZONAB, ZO-1-associated nucleic acid binding protein.

Aside from degrading ECM, MMP-10 is capable of digesting a broad spectrum of substrates harboring glutamate residue in P1 position. MMP-10 specifically cleaves multiple bioactive proteins that participate in cell adhesion, migration, proliferation, and differentiation, such as integrin α6 (ITGA6), cysteine-rich angiogenic inducer 61 (CYR61), and dermokine (DMKN) [13]. Recent studies have identified several novel MMP-10 substrates, such as HB-EGF, ZO-1, as well as pro-MMP-1, -7, -8, -9, -10, -13 [7,12,14,21], as shown in Figure 2. A detailed understanding of the molecular and cellular events involved in MMP-10 action is prerequisite for designing rational strategies for the therapeutic management of kidney diseases. Although activated MMP-10 has been observed inside the nuclei, intranuclear actions of MMP-10 have not been reported in the kidney up to date.

### 5.1. HB-EGF

HB-EGF is a membrane-anchored growth factor, which is widely expressed in glomerular and tubular compartments of adult kidneys at a very low level. In response to AKI, HB-EGF is markedly induced primarily in tubular epithelial cells [93]. As a unique epidermal growth factor receptor (EGFR) ligand, HB-EGF is activated post-translationally after cleavage of its ectodomain by diverse proteinases [94]. Activated HB-EGF then binds to EGFR and promotes renal epithelial cell repair, proliferation, and regeneration after injury [93,94]. Recent studies identify MMP-10 as the key enzyme responsible for mediating the cleavage of HB-EGF in human proximal tubular cells. As such, MMP-10 releases an active HB-EGF fragment capable of binding to EGFR, which prevents human proximal tubular epithelial cells from hypoxia-reoxygenation (H/R)-induced injury in vitro [12] (Figure 2A). The beneficial role of MMP-10 in AKI is in harmony with many earlier reports in which activation of EGFR signaling is vital for renal tubular injury repair and regeneration [95,96,97]. In agreement with this notion, the protective role of MMP-10 in AKI can be eradicated by erlotinib, a specific EGFR tyrosine kinase inhibitor [12,98].

Like EGFR, the role of the MMP-10/HB-EGF/EGFR axis in renal pathology is somehow contradictory and context-dependent, as both beneficial and deleterious effects have been reported in the kidney [99,100,101]. In addition to the protective role illustrated above, mounting evidence suggests that persistent tubule-specific EGFR activation contributes to renal fibrosis in response to chronic injury [102,103,104]. Furthermore, transgenic mice that selectively express human HB-EGF in the proximal tubule develop spontaneous, early onset, progressive tubulointerstitial fibrosis [105]. Mechanistically, activation of EGFR stimulates signal transduction and activator of transcription (STAT3), which interacts with the promoter region of homeodomain-interacting protein kinase 2 (*HIPK2*) gene and drives the progression of AKI to CKD [106]. These findings raise an intriguing possibility that MMP-10 facilitates tubulointerstitial fibrosis by cleaving HB-EGF, leading to activation of EGFR in the renal proximal tubule (Figure 2B).

### 5.2. ZO-1

ZO-1 is a tight junctional protein that constitutes the framework of podocyte junctions and plays an indispensable role in nephrogenesis and maintaining renal function [107,108,109]. The expression of ZO-1 in podocytes and its interaction with slit diaphragm components, such as nephrin, NEPH1 and NEPH3, are critical for maintaining the integrity of the filtration barrier of glomeruli [108,110]. A recent study indicates that MMP-10 specifically cleaves ZO-1, thereby impairing the integrity of slit diaphragm and resulting in podocyte dysfunction and proteinuria [7] (Figure 2C). This finding is in line with an earlier report demonstrating that specific loss of ZO-1 in podocytes leads to proteinuria and renal dysfunction with impaired glomerular filtration [111].

As a key tight junctional protein, ZO-1 has a function in modulating paracellular permeability by interacting with ZO-1-associated nucleic acid binding protein (ZONAB), a Y-box transcription factor, normally bound to the SH3 domain of ZO-1 at intercellular tight junctions [112,113,114] (Figure 2D). In this regard, ZO-1 might also function as an inhibitor of ZONAB [115]. The interaction between ZO-1 and ZONAB is essential for maintaining barrier function of epithelia and regulating epithelial cell proliferation and differentiation [116,117]. Destruction of ZO-1/ZONAB complex leads to nuclear translocation of ZONAB from tight junctions, resulting in target gene expression [118] (Figure 2D). 

Although prior work has focused primarily on the role of ZO-1 in slit diaphragm, it has been demonstrated that ZO-1 is also abundantly expressed in renal tubule epithelial cells [108,119,120]. The ZO-1/ZONAB signal pathway is indispensable for modulating epithelial phenotype. Certainly, following separation of ZONAB from ZO-1, nuclear ZONAB is positioned to shift the switch from differentiation to proliferation in proximal tubule epithelial cells in a cell density-dependent manner [121] (Figure 2D). Furthermore, ZONAB also promotes the stabilization and enhanced translation of p21 mRNA involved in cellular senescence [122,123]. In nephropathic cystinosis, overproduction of reactive oxygen species (ROS) contributes to the destruction of tight junction integrity and ZONAB nuclear translocation, which is disastrous for renal tubular epithelial function [115,124]. Based on these findings, it is tempting to speculate that the demolition of the tight junction and activation of a signaling cascade involving ZONAB in tubular cells, due to cleavage of ZO-1 by MMP-10, might be another mechanism in the evolution of CKD (Figure 2D). 

### 5.3. Other MMPs

As a proteolytic enzyme, MMP-10 is capable of cleaving pro-MMP-7 in a dose- and time-dependent manner, resulting in the release of mature MMP-7 [21]. Moreover, several direct lines of evidence suggest a role for MMP-10 in activating pro-MMP-9 in a dose-dependent manner, which rapidly produces four stable small fragments [13,21]. Of note, the proteolytic capacity of MMP-10 to pro-MMP-9 is more powerful than to pro-MMP-7. Recombinant pro-MMP-1, pro-MMP-8, as well as pro-MMP-13 could also be processed to the mature form by incubating with MMP-10 [3,14]. Of particularly interest, in contrast to a previous report that MMP-10 fails to directly digest pro-MMP-2 [21], a more recent study demonstrates that MMP-10 can cleave the collagen α2 (I) chain through activating an endogenous collagenolytic MMP with the molecular weight similar to activated MMP-2 [125]. Nevertheless, whether this collagenase is MMP-2 needs further investigation. 

The expression of MMP-1, MMP-2, MMP-7, MMP-8, MMP-9, MMP-10, as well as MMP-13 are upregulated after kidney damage [15,126]. Up to now, studies show all of them implicated in the pathophysiology of kidney disorders [1,127,128]. MMP-8 and MMP-13 have function in reducing kidney fibrosis by promoting matrix degradation after damage [129,130]. Contrary to this conclusion, there is a study showing that a selective MMP-13 inhibitor is able to alleviate renal fibrosis [131]. Taken together, these studies indicate that the ability of MMP-10 to super-activate other MMPs, including pro-MMP-1, pro-MMP-7, pro-MMP-8, pro-MMP-9, pro-MMP-10 and pro-MMP-13, might be another mechanism of MMP-10 involved in renal pathology (Figure 2E).

## 6. Conclusions and Future Perspectives

Over the past several years, there has been a growing interest in exploring the role of MMP-10 in kidney hemostasis and diseases. As discussed above, MMP-10 is upregulated in AKI, CKD, and RCC, primarily in renal tubular epithelia and glomerular podocytes. Functionally, MMP-10 elicits different cellular activities depending on the specific pathological condition and context. By cleaving HB-EGF, MMP-10 promotes tubular cell proliferation, repair and regeneration through EGFR signaling after AKI. In glomerular diseases, MMP-10 exacerbates proteinuria and glomerulosclerosis by impairing podocyte integrity through the cleavage of ZO-1. In RCC patients, dysregulated expression of MMP-10 is an independent risk factor for cancer invasion. Collectively, it becomes clear that MMP-10 not only plays a critical role in injury repair and tissue remodeling by degrading ECM components, but also affects a wide variety of biological processes through cleaving a broad spectrum of membrane-associated protein substrates.

Despite these progresses, our understanding of MMP-10 in the evolution of kidney disease is still limited. As activated MMP-10 is found inside the nuclei, the intranuclear actions of MMP-10 remain mysterious and await to be elucidated. In addition, although the present studies have provided enormous insights into the biological functions of MMP-10 in murine kidney diseases, there is still scarce evidence on the significance of MMP-10 in human AKI and CKD. Furthermore, since the roles of MMP-10 are extremely complex and context-dependent, how to translate the relevant knowledge into clinical therapy in patients is quite challenging and requires well-designed clinical trials. Because MMP-10 shares structural similarities with other MMPs and acts as an upstream regulator of several specific pathways involved in renal damage and repair, developing specific MMP-10 inhibitors with a favorable safety profile is needed. Undoubtedly, carefully addressing these issues will be instrumental for better understanding the role of MMP-10 in renal pathophysiology and for developing rational strategies of the treatment of kidney disorders.

## Figures and Tables

**Table 1:** Expression of MMP-10 in various kidney diseases.

Disease Model	Location	Expression	Ref.
Ischemic AKI ^1^	Renal tubular epithelia	Increase	[12,51]
Cisplatin-induced AKI	-	Increase	[12]
Rhabdomyolysis-associated AKI	-	Increase	[12]
ADR nephropathy ^2^	Glomerular podocyte	Increase	[7]
5/6 nephrectomy	-	Increase	[7]
T1DM ^3^	-	Increase	[49]
T2DM ^4^	Glomerular podocyte, juxtaglomerular apparatus	Increase	[7,50]
Alport syndrome	Glomerular podocyte	Increase	[33,52,53,54]
FSGS ^5^	Glomerular podocyte	Increase	[7,55]
IgA nephropathy	Glomerular podocyte	Increase	[7]
Renal cell carcinoma	Tubular cancer cells, sarcomatous cancer cell	Increase	[48]

^1^ Acute kidney disease, ^2^ Adriamycin nephropathy, ^3^ type 1 diabetes mellitus, ^4^ type 2 diabetes mellitus, ^5^ focal segmental glomerulosclerosis.

**Table 2:** Roles of MMP-10 in kidney diseases.

Disease Model	Intervention	Role	Ref.
IRI-induced AKI ^1^	Overexpression Knockdown	Promote tubular cell proliferation and inhibit apoptosis Aggravate AKI by promoting tubular cell apoptosis and inhibiting proliferation	[12]
Cisplatin-induced AKI	Overexpression	Alleviate tubular injury following nephrotoxic AKI	[12]
Non-diabetic geriatric AKI	-	Serve as a predictor for emergency renal replacement therapy (RRT)	[62]
DKD ^2^	Knockout	Protect against DKD, improve renal function, reduce mesangial expansion and macrophage influx	[49]
ADR-induced CKD ^3^	Overexpression Knockdown	Exacerbate podocyte injury and proteinuria Mitigate glomerular sclerosis and proteinuria	[7]
Hypertensive nephropathy	-	Contribute to the development of renal injury	[63]
RCC ^4^	Knockdown	Repress RCC invasion	[48,59]

^1^ Ischemia-reperfusion-induced acute kidney injury; ^2^ Diabetic kidney disease; ^3^ Adriamycin-induced chronic kidney disease; ^4^ Renal cell carcinoma.

## Data Availability

Not applicable.
